# Editorial – Déjà vu all over again

**DOI:** 10.1017/ehs.2021.51

**Published:** 2022-01-06

**Authors:** Ruth Mace

## Abstract

**Figure:**
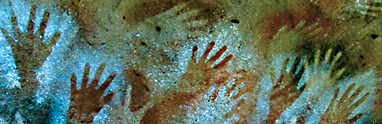

Writing this as Christmas approaches, I am completing my umpteenth Lateral Flow Test, cancelling flights as France closes its borders to Brits, and realising that the turkey I ordered for Christmas lunch is now looking somewhat on the large side. We have a horrible sense of déjà vu. While academic life was trying to get back to normal, and almost succeeding over the summer and autumn, we now have omicron strangling our social and professional lives. Despite the exasperation of this seemingly unending pandemic, I can't help feeling a certain scientific fascination at watching a disease evolve in real time, and how people gauge the risks, make decisions, assess their priorities and change their behaviour, often well ahead of any government instructions on how to behave.

However, our research goes on, and I am delighted you have continued to submit some of it to *Evolutionary Human Sciences*, despite this being the first year when we have had to charge an open access publication fee. We continue to publish on a diverse range of topics, ranging from object manipulation in wild orangutans (Schuppli, Van Cauwenberghe et al. 2021) to how related hunter–gatherers are to their group (Dyble et al., 2021) to what social contract theorists thought about human nature (Seabright et al., 2021) and cultural evolutionary insights to identify overlooked ‘hidden gems’ in the world of movies (Morin and Sobchuk, 2021; apparently including ‘Into the Wild’, which, if you somehow managed to overlook that one, is definitely a great suggestion for whiling away your time in covid-inflicted self-isolation). We celebrated the 150th anniversary of Darwin's *Descent of Man* with a special collection. Reading *Descent* (again or more likely for the first time) not only highlighted Darwin's amazing prescience, but also forced us to confront the horrible language used about race and sex in the nineteenth century. However, Darwin is not cancelled here, and we will celebrate the 150th anniversary of his ground-breaking ‘Expression of Emotions’ with another special collection this coming year. We are also building a collection on gendered conflict in the family, and another on evolutionary biomechanics and human locomotion. I am very excited about all of these. I am ever grateful to all our editors, authors, reviewers and publishers for their invaluable contributions of time and thought to our journal.

For 2022 I have decided to introduce a new category of paper, Perspectives. Several journals now have this format, but few are in our field. Perspectives articles enable the discussion of ideas from a personal viewpoint, more so than might be appropriate for a review or research paper. The aim is to air new ideas and opinions, challenge old ones and stimulate debate. That could be taken as an excuse to wax lyrical about hypotheses that are completely untestable (which is why I did not introduce this format sooner). It is nonetheless true that many personal ideas can be well worth airing, in ways that do not always fall into the remit of research reports or reviews. Apologies if you submitted something ‘perspectives-like’ before which I rejected because we did not have this format at that time. However, do contact me first before you submit, to give an idea of what the nature of your argument will be.

If you want your paper to be accepted, I just want to alert you to a few beefs of mine. I am not a fan of acronyms (even though I fully admit I may sometimes have used them). They annoy readers, mostly because we cannot quite remember what they mean, unless they are very simple and already familiar, but also they sometimes can solidify assumptions about categorisations or processes that cannot be made (like WEIRD, which encourages us to simplify cultural diversity into an unacceptably simplistic dichotomy; Ghai, 2021). It can sound a bit presumptive to make up a name for your own hypothesis. If your theory becomes useful, I am sure someone will name it for you. In research papers, there is no need to say you are the first person to say something; you may or may not be, but more importantly, thinking that only the first person to do something is making scientific progress does not help with the replication crisis. If we only publish the first attempt, it is no wonder we will sometimes get the wrong answer. I am more than happy to publish formal or informal replications. I do interpret our brief very widely: ‘Evolutionary’ means all kinds of things, from function to archaeology, and even ‘Human’ is a category I am happy to stretch – primates are definitely all in, and we are now handling a paper about dogs and quite willing to look more widely if appropriate. And ‘Sciences’? Tricky, but again I am not a purist.

This time last year I said I hoped that vaccines would make 2021 different from 2020. They have gone a long way to doing so, at least for some of us. I am forever grateful to be in a country where I can benefit from scientific advances, and hopefully the next round of vaccines will do even more. Yet vaccines alone cannot do it all it seems. There are now four stockings hanging at my fireplace, all intended for those in the 20–29 age group from London, one of whom said ‘Nice, but optimistic …’. Stay optimistic, and I wish you all a wonderful Christmas (if you do that) and a very happy New Year.
